# Endoscopically observable white nodule caused by distal intramural lymphatic spread of rectal cancer: a case report

**DOI:** 10.1186/1477-7819-10-216

**Published:** 2012-10-11

**Authors:** Ayako Tsumura, Shozo Yokoyama, Katsunari Takifuji, Tsukasa Hotta, Kenji Matsuda, Takashi Watanabe, Yasuyuki Mitani, Hiroki Yamaue

**Affiliations:** 1 Second Department of Surgery, Wakayama Medical University, School of Medicine, 811-1 Kimiidera, Wakayama 641-8510, Japan

**Keywords:** Rectal cancer, White nodule, Intramural spread, Lymphatic permeation

## Abstract

This report describes a case of rectal cancer with endoscopically observable white nodules caused by distal intramural lymphatic spread. A 57-year-old female presented to our hospital with frequent diarrhea and hemorrhoids. Computed tomography showed bilateral ovarian masses and three hepatic tumors diagnosed as rectal cancer metastases, and also showed multiple lymph node involvement. The patient was preoperatively diagnosed with stage IV rectal cancer. Colonoscopy demonstrated that primary rectal cancer existed 15 cm from the anal verge and that there were multiple white small nodules on the anal side of the primary tumor extending to the dentate line. Biopsies of the white spots were performed, and they were identified as adenocarcinoma. The patient underwent Hartmann’s procedure because of the locally advanced primary tumor. The white nodules were ultimately diagnosed as being caused by intramural lymphatic spreading because lymphatic permeation was strongly positive at the surrounding area. Small white nodules near a primary rectal cancer should be suspected of being intramural spreading. Endoscopic detection of white nodules may be useful for the diagnosis of distal intramural spread.

## Background

To avoid local recurrence of rectal cancer, it is important to determine an appropriate distance of the distal resection margin of the tumor. Distal intramural spread is one of the crucial factors involved in making the decision about the extent of the anal resection margin [1-5]. Transrectal ultrasonography has been reported to be useful for preoperative detection of distal intramural spread [6]. However, there are no reports concerning endoscopic detection of distal intramural metastasis. Here we present the case of a patient with rectal cancer who had endoscopically observable white nodules caused by distal intramural lymphatic spread.

## Case presentation

**Figure 1:**
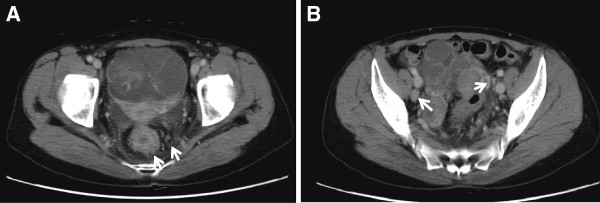
A Primary tumor with rectal wall thickness and swollen lymph node around the tumor. B Swollen lateral lymph nodes.

**Figure 2:**
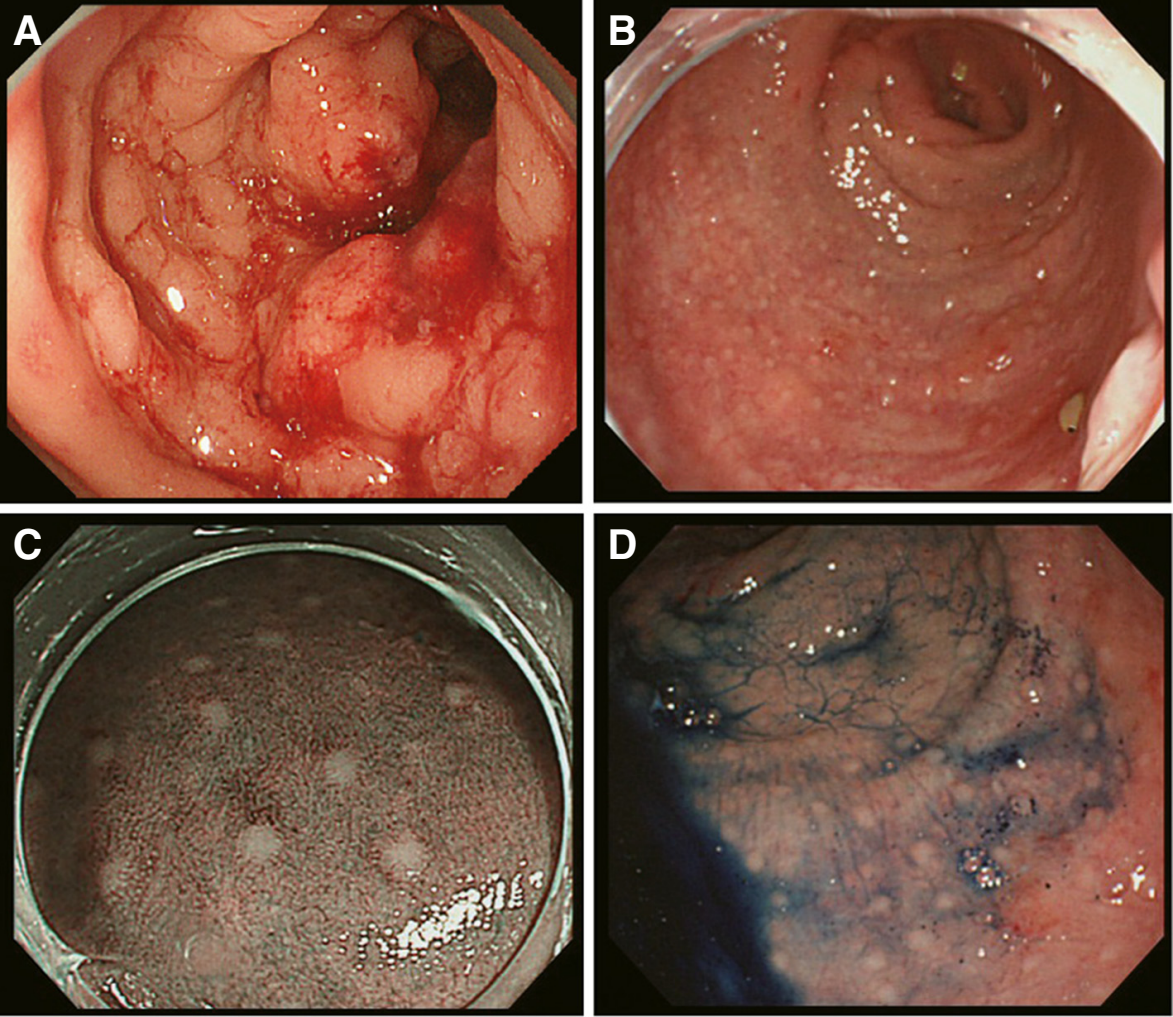
A Primary tumor located 15 cm from the anal verge at preoperative colonoscopy. B Small nodules existed on the anal side of the main tumor and irregular mucosal surface associated with intramural spread of cancer. C Narrow band imaging of small nodules. D Endoscopic examination with indigo carmine.

A 57-year-old female presented with frequent diarrhea and hemorrhoids. Computed tomography (CT) showed bilateral ovarian masses (right: 38 mm, left: 87 mm) and three hepatic tumors diagnosed as rectal cancer metastases; multiple upper lymph node involvement including the superior rectal artery region and the inferior mesenteric artery region; and lateral lymph node involvement including the internal iliac, common iliac, and external iliac artery regions (Figure 1). Fluorodeoxyglucose positron emission tomography (FDG-PET) demonstrated that the standardized uptake value (SUV) maximum was 2.64 at the main tumor and 4.21 at the ovarian mass. Some blood tumor markers were increased: carcinoembryonic antigen, 16.9 ng/ml; carbohydrate antigen 19–9, 20.0 U/ml; carbohydrate antigen 125, 40.0 U/ml. The patient was preoperatively diagnosed with stage IV rectal cancer. Colonoscopy revealed that the tumor was located 15 cm from the anal verge, the irregular mucosal surface was associated with intramural spread of cancer, and multiple white small nodule-like lymphoid follicles existed on the anal side of the primary tumor extending to the dentate line (Figure 2).

**Figure 3:**
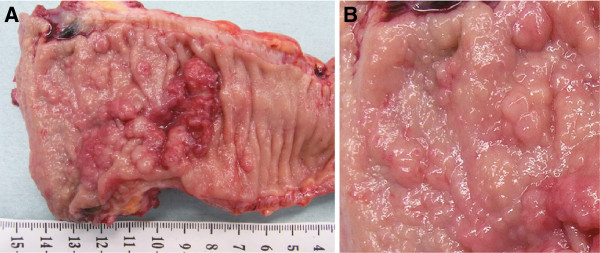
A 36 × 29-mm tumor in the rectum and small nodules. B Small nodules with smooth surfaces.

Histological examination of the biopsy from a white spot near the dentate line specimen showed moderately differentiated adenocarcinoma. Therefore, abdominoperineal resection was planned for the patient. However, because of peritoneal metastasis, the patient underwent Hartmann’s procedure. The primary tumor was 36 × 29 mm in diameter with a positive distal margin and circumferential resection margin (Figure 3A). Multiple small nodules (1–6 mm) existed on the anal side of the main tumor. The nodules’ surfaces were smooth like lymphoid follicles (Figure 3B).

**Figure 4:**
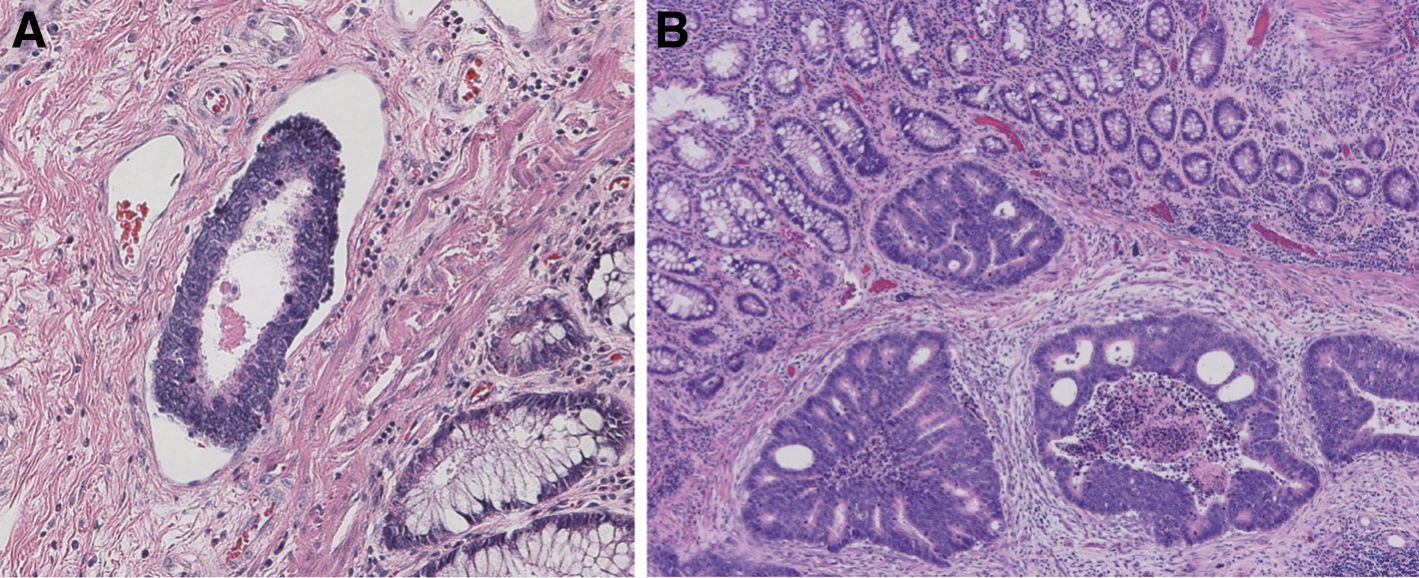
A Hematoxylin and eosin staining revealing severe lymphovascular invasion. B Cancer cells existed in the submucosa and muscularis mucosae layer, but not in the mucosa.

The pathological findings revealed moderately differentiated adenocarcinoma invading the serosa, which was exposed with metastatic lymph nodes. Lymphatic permeation was strongly positive, and the cancer cells formed a small mass (Figure 4A). The small white nodules observed on the anal side of the tumor were moderately differentiated adenocarcinoma existing in the lymphatic vessels of the submucosa and muscularis mucosae, but not on the mucosa (Figure 4B). The patient received eight courses of chemotherapy (XELOX + bevacizumab; capecitabine 2,000 mg/m^2^/day, days 1–14; oxaliplatin 130 mg/m^2^, day 1; bevacizumab 7.5mg/kg, day 1) after surgery, and then the metastatic hepatic tumors shrank. The patient is doing well at 12 months after surgery.

## Discussion

The local recurrence rate of rectal cancer patients after surgery with total mesorectal excision is reported to be 6–9.7% [7-9]. To reduce local recurrence, one of the important points is to determine the appropriate distal margin. Some reports have shown that distal intramural spread was observed in 10.6-40% of rectal cancer [1-5,10,11]. Some reports have shown that 2 cm distance of the distal resection margin from the tumor is safe, since distal intramural spreading rarely exceeds 2 cm [2,10,11]. The rate of intramural spread exceeding 1 cm in rectal cancer cases is 10% [11], and more than 2 cm was reported in 1.3-6% of rectal cancer cases [1,2,10,11]. So far, distal intramural spread is determined by pathological findings; therefore, it cannot be diagnosed prior to surgery. If preoperative detection of intramural spread becomes possible, it will be helpful for reducing the local recurrence rate.

The pattern of metastases to the anus is implanted or lymphovascular metastases [12]. Implantation of rectal carcinoma cells has often been reported in the last half century. Rectal cancer cells are able to metastasize to the anal fistula [13,14], biopsy wounds [15], suture lines [16-18], and obstructive colitis [19]. Lymphovascular metastasis in the literature is defined as anorectal metastatic cancer with an intact anorectal epithelium [12]. In this case, the histological findings showed that small white nodules, consisting of cancer cells, existed in the lymphatic vessels of the submucosa and muscularis mucosae, but not on the mucosa (Figure 4B). Therefore, these white spots appeared to metastasize via microlymphatic ducts and invaded the submucosa. Rectal carcinoma is able to invade and metastasize in other ways, such as direct invasion and hematogenous spread. In the present case, direct extension, intraluminal implantation, and hematogenous spread did not occur, as shown by the discontinuous spread of the tumor, no cancer cells on the mucosa, and the smaller number of venous permeations, respectively. Finally, we determined that the white nodules were caused by lymphatic invasion.

Local recurrence by lymphovascular metastasis to the anus has been shown to be uncontrollable despite wide resection [12]. The presence of lymphovascular invasion as an independent factor for local recurrence in a multivariate analysis has been reported [20], implying that distal intramural lymphatic metastasis to the anal side is an important factor for local recurrence. Lymphatic spreading is characterized by massive invasion into lymphatic vessels and lymph node metastases. Therefore, excision of the primary lesion and the usual resected distal margin of the rectum are insufficient for patients with massive distal intramural lymphatic spread. Radical surgery including the metastasized lymphatic system may be required to achieve better local control. Prior to surgery, if colonoscopy reveals white nodules on the anal side of the tumor, radical surgery should be performed to avoid local recurrence.

## Conclusion

This report presented a case of rectal cancer with white nodules on the anal side of the tumor. The white nodules were caused by distal intraluminal lymphatic spread. Endoscopic diagnosis of white nodules around the tumor may reduce local recurrence of rectal cancer.

## Consent

Written consent was obtained from the patient for publication of this study and the related photos.

## Competing interests

The authors declare that they have no competing interest**s**.

## Authors contributions

AT performed the surgery, literature search, and write up of the manuscript. SY initiated the concept, performed the surgery, helped in the literature search, and gave approval for the final write up. KT performed the surgery. TH helped in the revision of the article. KM helped in the revision of the article. TW helped in the revision of the article. YM helped in the revision of the article. HY contributed to the clinical management of the patient and helped in the revision of the article. All authors read and approved the final manuscript.

## Authors information

**Writing assistance:** Dr. Brian Quinn, Editor-in-Chief, Japan Medical Communication.
